# The estimated glomerular filtration rate predicts pacemaker-induced cardiomyopathy

**DOI:** 10.1038/s41598-023-43953-7

**Published:** 2023-10-02

**Authors:** Mitsunori Oida, Eriko Hasumi, Goto Kohsaku, Kani Kunihiro, Tsukasa Oshima, Takumi J. Matsubara, Jun Matsuda, Yu Shimizu, Gaku Oguri, Toshiya Kojima, Katsuhito Fujiu, Issei Komuro

**Affiliations:** 1 Department of Cardiovascular Medicine, Graduate School of Medicine, The University of Tokyo, 7-3-1 Hongo, Bunkyo, Tokyo, 113-8655 Japan; 2 Department of Advanced Cardiology, Graduate School of Medicine, The University of Tokyo, Tokyo, Japan

**Keywords:** Cardiology, Cardiac device therapy, Kidney diseases

## Abstract

Clinical predictors for pacemaker-induced cardiomyopathy (PICM) (e.g., a wide QRS duration and left bundle branch block at baseline) have been reported. However, factors involved in the development of PICM in patients with preserved left ventricular ejection fraction (LVEF) remain unknown. This study aimed to determine the risk factors for PICM in patients with preserved LVEF. The data of 113 patients (average age: 71.3 years; men: 54.9%) who had echocardiography before and after pacemaker implantation (PMI) among 465 patients undergoing dual-chamber PMI were retrospectively analyzed. Thirty-three patients were diagnosed with PICM (18.0/100 person-years; 95% CI 12.8–25.2). A univariate Cox regression analysis showed that an estimated glomerular filtration rate (eGFR) ≤ 30 mL/min/1.73 m^2^ (HR 3.47; 95% CI 1.48–8.16) and a past medical history of coronary artery disease (CAD) (HR 2.76; 95% CI 1.36–5.60) were significantly associated with the onset of PICM. After adjusting for clinical variables, an eGFR ≤ 30 mL/min/1.73 m^2^ (HR 2.62; 95% CI 1.09–6.29) and a medical history of CAD (HR 2.32; 95% CI 1.13–4.80) were independent risk factors for developing PICM. A medical history of CAD and low eGFR are independent risk factors for PICM in patients with preserved LVEF at baseline. These results could be helpful in predicting a decreased LVEF by ventricular pacing before PMI. Close follow-up by echocardiography is recommended to avoid a delay in upgrading to physiological pacing, such as cardiac resynchronization therapy or conduction system pacing.

## Introduction

Chronic right ventricular (RV) pacing sometimes leads to a deterioration in the left ventricular ejection fraction (LVEF) because RV pacing provokes interventricular dyssynchrony and intraventricular dyssynchronous contraction in the left ventricle (LV)^1^. A combination of electrical and mechanical dyssynchrony leads to adverse LV remodeling, which promotes heart failure (HF), atrial fibrillation (AF), and mortality^2–4^.

A deterioration in LV systolic function related to pacemaker implantation (PMI) is termed pacemaker-induced cardiomyopathy (PICM)^5,6^. Previous studies have used different LVEF thresholds to define PICM. Therefore, there is currently no internationally accepted definition of PICM. The following three definitions of PICM have been used in past clinical studies: (1) an LVEF ≤ 40% if the baseline LVEF is ≥ 50% or an absolute reduction in the LVEF ≥ 5% if the baseline is < 50%; (2) an LVEF ≤ 40% if the baseline LVEF is ≥ 50% or an absolute reduction in the LVEF ≥ 10% if the baseline is < 50%; and (3) an absolute reduction in the LVEF ≥ 10%, regardless of baseline LV function^2,7–9^.

There is wide variation in the published prevalence of the development of PICM (5–27%)^10,11^. The highest prevalence was reported to be 39% according to the definition of an absolute reduction in the LVEF ≥ 10% during a follow-up of 3–4 years^2^. Furthermore, a worldwide survey showed that the number of patients undergoing PMI had increased^12^. Therefore, the number of patients with PICM should also be increased.

Identifying risk factors for PICM before PMI is beneficial for preventing PICM-associated heart diseases, such as HF. It is reported that an older age^6,7,9^, male sex^7,13^, a history of myocardial infarction^13^, a lower baseline LVEF^14,15^, a wider intrinsic QRS duration^7,13,16^, left bundle branch block (LBBB)^13^, a history of AF^6,17^, and a chronic higher RV pacing burden^8^ could be risk factors for PICM. However, the precise mechanism and established risk factors for PICM have not been fully identified^18^. Therefore, the present study aimed to identify additional risk factors for PICM.

## Methods

### Definition of PICM

In this study, PICM was defined as follows: (1) exclusion of alternative causes of heart diseases, such as acute myocardial ischemia, uncontrollable tachyarrhythmia, frequent premature contractions, and untreated hypertension; and (2) an absolute reduction in the LVEF ≥ 10% as measured by transthoracic echocardiography (TTE) after PMI compared with before PMI. TTE data were collected within six months before PMI and from three months to three years after PMI. All TTE data were over-read by cardiovascular experts.

### Data collection and the study population

**Figure 1:**
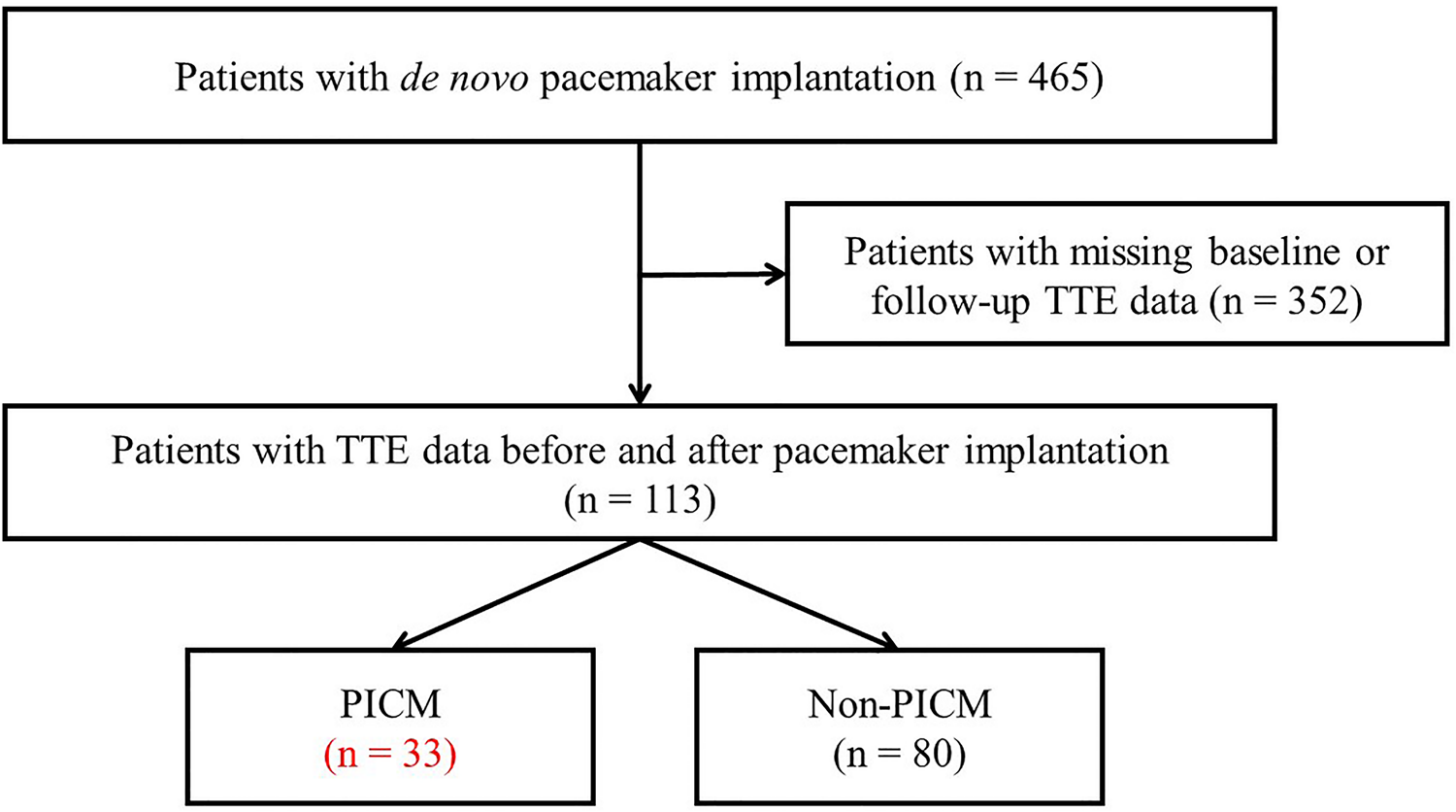
Flowchart of data collection for the study population. Patients who underwent de novo pacemaker implantation were studied between December 2006 and November 2018. Among them, 113 patients who matched the criteria were enrolled. *TTE* transthoracic echocardiogram, *PICM* pacing-induced cardiomyopathy.

All data were retrospectively acquired at the University of Tokyo Hospital, Tokyo, Japan, from patients who underwent de novo PMI between December 2006 and November 2018. PMI was performed in patients with sick sinus syndrome (SSS) or atrioventricular block (AVB). A total of 465 patients undergoing PMI were included in this study. To identify the risk factors for PICM, the medical history and clinical data were retrospectively collected from all patients. The flow of data collection for the study is shown in Fig. 1.

The inclusion criteria were as follows: (1) age of ≥ 20 years; (2) de novo PMI; and (3) available data of TTE before and after PMI. The exclusion criteria were as follows: (1) implantation of a prior cardiac implantable electronic device; (2) lack of TTE before and/or after PMI; (3) congenital heart diseases; (4) heart transplantation; and (5) alternative cause of a reduction in the LVEF, such as acute myocardial ischemia, valvular heart disease, uncontrollable tachyarrhythmia, frequent premature contractions, and untreated hypertension. In addition, all of the patients’ laboratory data were obtained upon admission to our hospital and at a follow-up LVEF examination after discharge.

### Clinical outcome and definition of clinical variables

The primary outcome was an incidence of a reduced ejection fraction, which was defined as a ≥ 10% reduction in the LVEF in a follow-up TTE after PMI. The New York Heart Association (NYHA) class was categorized on the basis of symptoms and evaluation of a medical examination on admission by expert cardiologists. AF was diagnosed by an electrocardiogram on admission. Diabetes mellitus was diagnosed as a glycated hemoglobin value ≥ 6.5% or determined by a history of taking oral hypoglycemic agents or insulin injections. Hypertension (HT) was diagnosed as a systolic blood pressure ≥ 140 mmHg or diastolic blood pressure ≥ 90 mmHg. A history of antihypertensive agents was also used to determine HT. The estimated glomerular filtration rate (eGFR) was calculated as 194 × (serum creatinine)^−1.094^ × (age)^−0.287^ × 0.739 (for female patients)^19^. Chronic kidney disease (CKD) was defined as an eGFR < 60 mL/min/1.73 m^2^.

### Statistical analysis

The patients were divided into two groups, the PICM group and the non-PICM group, on the basis of the definition of PICM as previously described. The differences in baseline characteristics were compared between the two groups by Student’s *t*-test or Mann–Whitney *U* test for continuous variables and the Chi-square test for categorical variables, as appropriate. The incidence rate of PICM and its 95% confidence interval (CI) were calculated in person-years with the assumption of Poisson distribution for the total patients. The cumulative rates of PICM were calculated with the Kaplan–Meier method, and time-to-event data are shown in Kaplan–Meier curves. Cox univariate and multivariate analyses were performed to estimate hazard ratios (HRs) of clinical variables for developing PICM. All statistical analyses were performed using IBM SPSS version 28.0.0.0 (IBM Corp., Armonk, NY), and a two-tailed *P* value < 0.05 indicated statistical significance.

### Ethical approval

This study was approved by the University of Tokyo institutional ethics committee (approval number 2650-[13]). All patient information was deidentified and the requirement for written informed consent was waived by the University of Tokyo institutional ethics committee. The study protocol was conducted in accordance with the Declaration of Helsinki.

## Results

### Clinical and procedural characteristics

One hundred and thirteen patients who underwent de novo PMI and had TTE data before and after PMI were enrolled. The patients’ characteristics are shown in Table 1. The mean age was 71.3 ± 11.2 years old (range: 25–91 years), the mean LVEF before PMI was 65.0 ± 13.0%, and the mean eGFR before PMI was 59.3 ± 24.8 mL/min/1.73 m^2^. All patients underwent implantation of the dual-chamber pacemakers, and 77 (68.1%) underwent PMI for SSS and 36 (31.9%) for AVB. Of the 36 patients diagnosed with atrioventricular block (AVB), the following classifications were observed: 26 patients, or 72.2%, exhibited complete AVB; six patients, representing 16.7%, had 2:1 AVB; and the remaining four patients, accounting for 11.1%, were diagnosed with advanced AVB.

**Table 1:** The characteristics of patients.

Characteristics	Total (n = 113)	Non-PICM (n = 80)	PICM (n = 33)	*P*
Demographics				
Age (years)	71.3 ± 11.2	71.8 ± 10.4	70.0 ± 13.2	0.44
Male sex (n, %)	62 (54.9)	42 (52.5)	20 (60.6)	0.28
BMI (kg/m^2^)	22.9 ± 3.8	22.6 ± 3.9	23.4 ± 3.5	0.32
Echocardiography				
LVEF (%)	65.0 ± 13.0	63.7 ± 11.8	68.0 ± 15.2	0.11
LVDd (mm)	47.2 ± 7.1	47.1 ± 6.8	47.7 ± 7.7	0.66
LVDs (mm)	30.2 ± 7.4	30.5 ± 7.4	29.5 ± 7.2	0.51
LAD (mm)	42.8 ± 8.7	42.9 ± 9.0	42.6 ± 8.1	0.81
Medical history and clinical findings				
CAD (n, %)	44 (38.9)	26 (32.5)	18 (54.5)	0.03*
CABG	8 (7.1)	5 (6.3)	3 (9.1)	0.69
PCI	33 (29.2)	14 (42.4)	19 (23.8)	0.05*
DM (n, %)	39 (34.5)	26 (32.5)	13 (39.4)	0.31
HT (n, %)	74 (65.5)	55 (68.8)	19 (57.6)	0.18
HF (n, %)	33 (29.2)	23 (28.7)	10 (30.3)	0.52
NYHA class ≥ II, n (%)	52 (46.0)	36 (45.0)	16 (48.5)	0.45
Arrythmia and ECG findings				
AF (n, %)	48 (42.5)	34 (42.5)	14 (42.4)	0.58
AVB indicating PMI (n, %)	36 (31.9)	23 (28.7)	13 (39.4)	0.19
RBBB (n, %)	23 (20.4)	16 (20.0)	7 (21.2)	0.54
LBBB (n, %)	10 (8.8)	10 (12.5)	0 (0)	0.03*
QRS duration (ms)	113.5 ± 24.4	116.9 ± 25.6	105.2 ± 19.1	0.02*
Kidney function				
eGFR (mL/min/1.73 m^2^)	59.3 ± 24.8	62.7 ± 23.9	50.9 ± 25.2	0.02*
eGFR ≥ 90 (n, %)	7 (6.2)	5 (6.3)	2 (6.1)	0.67
eGFR ≥ 60 and < 90 (n, %)	52 (46.0)	40 (50.0)	12 (36.4)	0.13
eGFR < 60 (n, %)	54 (47.8)	35 (43.8)	19 (57.6)	0.13
eGFR ≤ 30 (n, %)	13 (11.5)	5 (6.3)	8 (24.2)	0.01*

*PICM* pacemaker-induced cardiomyopathy, *BMI* body mass index, *LVEF* left ventricular ejection fraction, *LVDd* left ventricular end-diastolic diameter, *LVDs* left ventricular end-systolic diameter, *LAD* left atrial diameter, *CAD* coronary artery disease, *PCI* percutaneous coronary intervention, *CABG* coronary artery bypass graft, *DM* diabetes mellitus, *HT* hypertension, *HF* heart failure, *NYHA* New York Heart Association, *AF* atrial fibrillation, *AVB* atrioventricular block, *RBBB* right bundle branch block, *LBBB* left bundle branch block, *eGFR* estimated glomerular filtration rate. *P < 0.05.

Among the enrolled patients, 44 (38.9%) had a medical history of coronary artery disease (CAD), and those of 33 (29.2%) patients had undergone percutaneous coronary intervention (PCI). Patients in the PICM group had a more prevalent medical history of CAD (54.5% vs. 32.5%, *P* = 0.03) and a history of previous PCI (42.4% vs. 29.2%, *P* = 0.05), lower eGFR (50.9 ± 25.2 mL/min/1.73 m^2^ vs. 62.7 ± 23.9 mL/min/1.73 m^2^, *P* = 0.02), higher rate of eGFR ≤ 30 mL/min/1.73 m^2^ (24.2% vs. 6.3%, *P* = 0.01), lower rate of LBBB (0% vs. 12.5%, *P* = 0.03), and shorter QRS duration (105.2 ± 19.1 ms vs. 116.9 ± 25.6 ms, *P* = 0.02) than the non-PICM group. There were no significant differences in other background factors between the two groups.

Supplementary Table 1 outlines the positions of the RV lead tips. The distribution of these lead tips across various regions is as follows: apex with 32 patients (28.3%), septum with 70 patients (61.9%), left bundle area with 3 patients (2.7%), and His bundle area with 8 patients (7.1%). It is noteworthy that there were no significant disparities in the frequency of RV lead placement at the RV apex between patients diagnosed with PICM and those without.

Right atrial (RA) and RV pacing frequencies in patients in the PICM and non-PICM groups after PMI were investigated retrospectively. Pacemaker interrogation data were obtained at the closest date of the TTE examination. There were no significant differences in RA or RV pacing frequencies between the two groups (Table 2).

**Table 2:** Right atrial and right ventricular pacing frequencies.

Characteristics	Total (n = 113)	Non-PICM (n = 80)	PICM (n = 33)	*P*
RAp (%)	40.2 ± 38.5	38.9 ± 38.8	43.4 ± 38.3	0.58
RVp (%)	52.9 ± 44.8	49.7 ± 44.9	60.4 ± 44.5	0.25

*PICM* pacemaker-induced cardiomyopathy, *RAp* right atrial pacing, *RVp* right ventricular pacing.

### Prevalence of PICM

**Figure 2:**
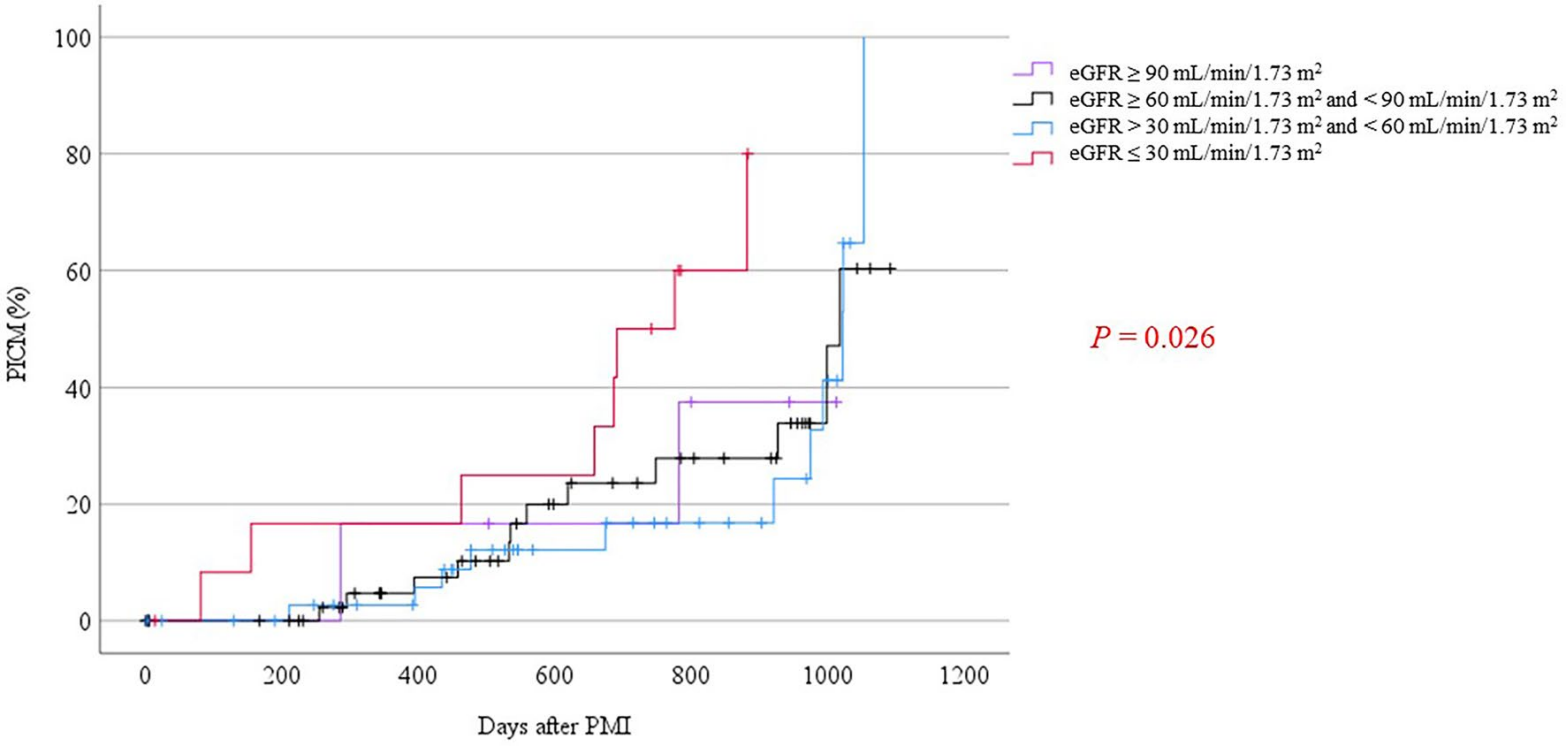
Incidence of PICM in patients with chronic kidney disease. Kaplan–Meier curves are shown for the development of PICM over 3 years of follow-up. The eGFR was categorized into four groups: eGFR ≥ 90 mL/min/1.73 m^2^, eGFR ≥ 60 mL/min/1.73 m^2^ and < 90 mL/min/1.73 m^2^, eGFR > 30 mL/min/1.73 m^2^ and < 60 mL/min/1.73 m^2^, and eGFR ≤ 30 mL/min/1.73 m^2^. A significant difference in the incidence of PICM among the four groups was detected. *PICM* pacing-induced cardiomyopathy, *eGFR* estimated glomerular filtration rate (mL/min/1.73 m^2^).

**Figure 3:**
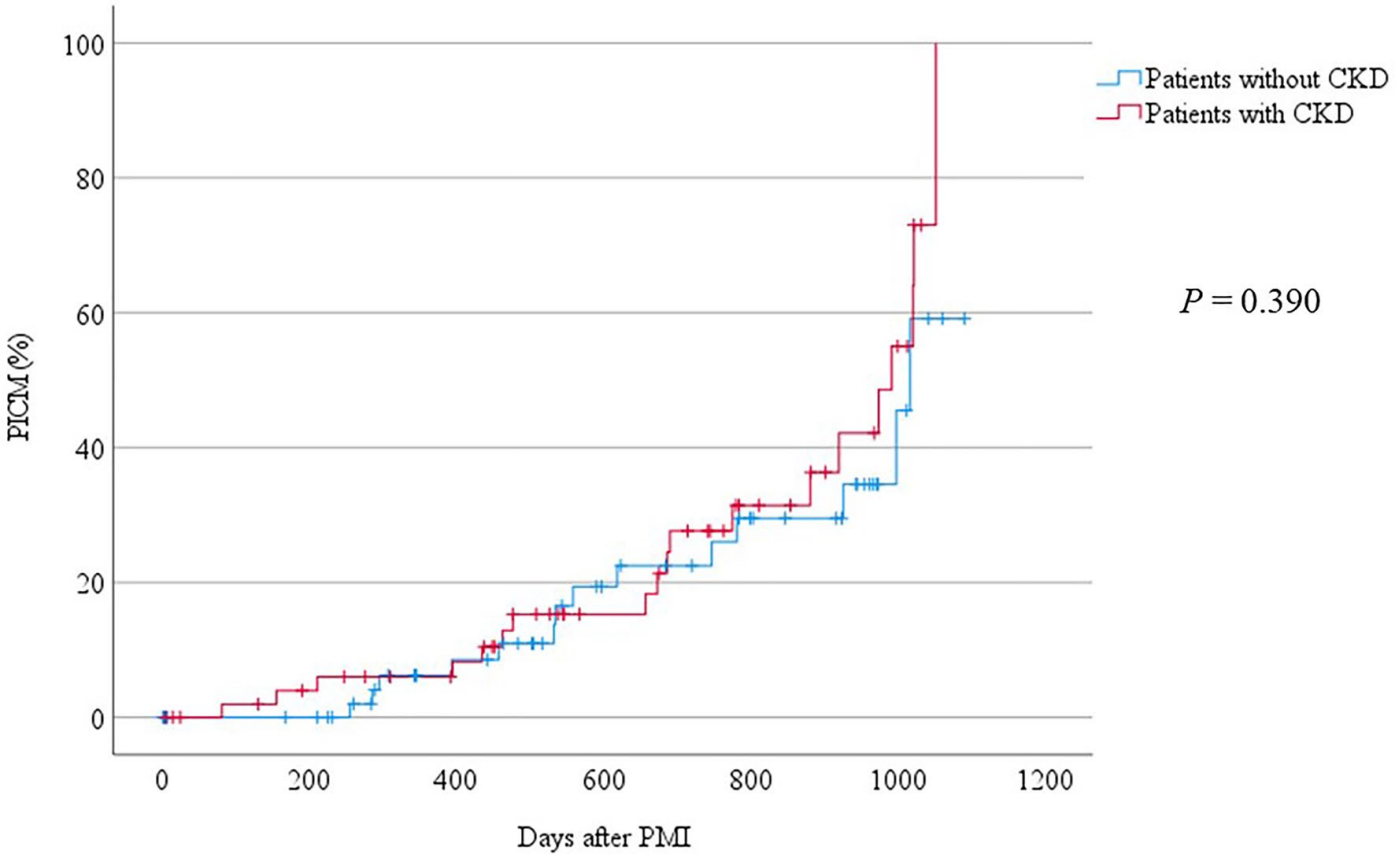
Incidence of PICM in patients with CKD with an eGFR < 60 mL/min/1.73 m^2^. Kaplan–Meier curves are shown for the development of PICM. The log-rank test was used for comparing patients with and without CKD. There was no significant difference in the incidence of developing PICM between the two groups. *PICM* pacing-induced cardiomyopathy, *CKD* chronic kidney disease.

During a mean follow-up of 1.6 ± 0.9 years, 33 (29.2%) patients developed PICM, and the incidence rate was 18.0/100 person-years (95% CI 12.8–25.2) in the total follow-up of 184 person-years. Kaplan–Meier curves for the rates of developing PICM are shown in Figs. 2 and 3. These rates were evaluated with the log-rank test for determining differences between patients with and without CKD. In addition, these patients were divided into four groups by the eGFR as follows: (A) eGFR ≥ 90 mL/min/1.73 m^2^, (B) eGFR ≥ 60 mL/min/1.73 m^2^ and < 90 mL/min/1.73 m^2^, (C) eGFR > 30 mL/min/1.73 m^2^ and < 60 mL/min/1.73 m^2^, and (D) eGFR ≤ 30 mL/min/1.73 m^2^. There was a significant difference in the incidence of PICM between the four groups by the eGFR (log-rank, *P* = 0.03). Additionally, there were marked differences in the incidence of PICM between groups B and D, as well as between groups C and D, with log-rank values of P = 0.02 and P < 0.01, respectively. These findings are detailed in Table 3. Remarkably, patients with an eGFR < 30 mL/min/1.73 m^2^ showed a higher incidence of PICM than those with an eGFR ≥ 30 mL/min/1.73 m^2^.

**Table 3:** Log-rank analysis for incidence of pacemaker-induced cardiomyopathy based on renal function status.

eGFR	*P*
eGFR ≥ 90 mL/min/1.73 m^2^ (control)	
eGFR ≥ 60 mL/min/1.73 m^2^ and < 90 mL/min/1.73 m^2^	0.90
eGFR > 30 mL/min/1.73 m^2^ and < 60 mL/min/1.73 m^2^	0.64
eGFR ≤ 30 mL/min/1.73 m^2^	0.21
eGFR ≥ 60 mL/min/1.73 m^2^ and < 90 mL/min/1.73 m^2^ (control)	
eGFR > 30 mL/min/1.73 m^2^ and < 60 mL/min/1.73 m^2^	0.90
eGFR ≤ 30 mL/min/1.73 m^2^	0.02*
eGFR > 30 mL/min/1.73 m^2^ and < 60 mL/min/1.73 m^2^ (control)	
eGFR ≤ 30 mL/min/1.73 m^2^	< 0.01**

*eGFR* estimated glomerular filtration rate. *P < 0.05; **P < 0.01.

### Risk factors for PICM

A univariate Cox regression analysis of clinical features that were associated with the prediction of PICM was performed (Table 4**)**. Every 10 mL/min/1.73 m^2^ decrease in the eGFR (HR 1.19, 95% CI 1.01–1.40), an eGFR ≤ 30 mL/min/1.73 m^2^ (HR 3.47, 95% CI 1.48–8.16), and a medical history of CAD (HR 2.76, 95% CI 1.36–5.60) were significantly related to the development of PICM. However, neither LBBB nor every 10-ms prolongation of the QRS duration was significantly associated with PICM (HR 0.86, 95% CI 0.73–1.01; HR 1.19, 95% CI 1.01–1.40, respectively).

**Table 4:** Univariate Cox model of hazard ratios for pacemaker-induced cardiomyopathy.

Variables	HR	95% CI	*P*
Every 10-year increase in age	0.97	0.72–1.31	0.83
Male sex	1.00	0.50–2.01	1.00
Every 10% decrease in the baseline LVEF	0.98	0.70–1.36	0.90
Every 10-ms prolongation of the QRS duration	0.86	0.73–1.01	0.07
LBBB	0.05	0.00–170.00	0.46
History of AF	0.84	0.42–1.69	0.63
Every 10 mL/min/1.73 m^2^ decrease in eGFR	1.19	1.01–1.40	0.04*
eGFR ≤ 30 mL/min/1.73 m^2^	3.47	1.48–8.16	< 0.01**
CAD	2.76	1.36–5.60	< 0.01**

*HR* hazard ratio, *CI* confidence intervals, *LVEF* left ventricular ejection fraction, *LBBB* left bundle branch brock, *AF* atrial fibrillation, *eGFR* estimated glomerular filtration rate, *CAD* coronary artery disease. *P < 0.05; **P < 0.01.

**Figure 4:**
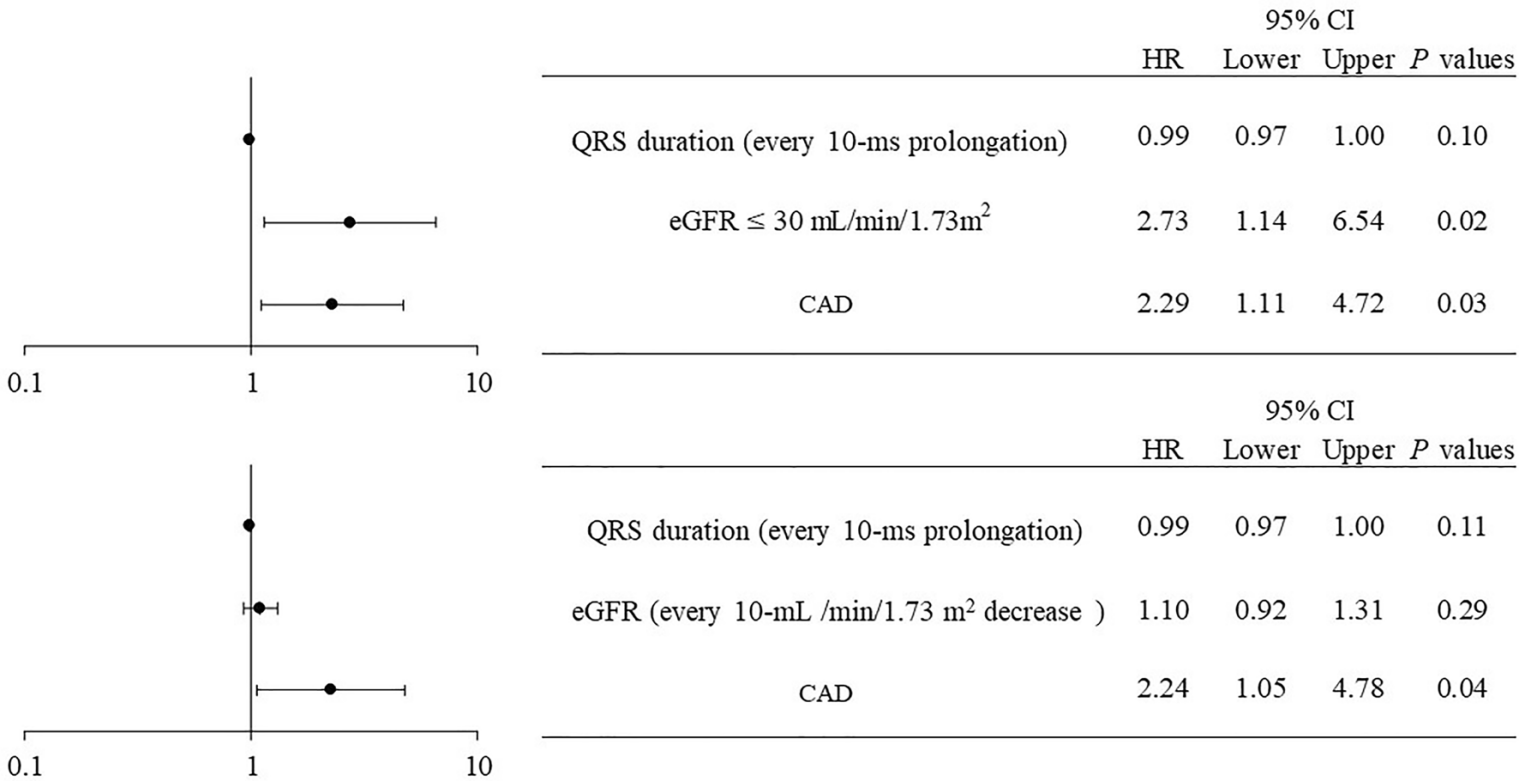
Forest plot of hazard ratios for PICM according to a multivariate analysis by the Cox model. After adjusting for the QRS duration, eGFR, and CAD, a multivariate Cox regression analysis showed that an eGFR ≤ 30 mL/min/1.73 m^2^ and CAD were independent risk factors for developing PICM. *CI* confidence interval, *HR* hazard ratio, *eGFR* estimated glomerular filtration rate, *CAD* coronary artery disease.

After adjusting for every 10-ms prolongation of the QRS duration, an eGFR ≤ 30 mL/min/1.73 m^2^, and a medical history of CAD, the multivariate Cox regression analysis showed that an eGFR ≤ 30 mL/min/1.73 m^2^ and CAD were significant independent risk factors for PICM (HR 2.62, 95% CI 1.09–6.29, and HR 2.32, 95% CI 1.13–4.80, respectively) (Fig. 4).

## Discussion

Previous studies have reported that an older age^6,7,9^, male sex^7,13^, a history of myocardial infarction^13^, a lower baseline LVEF^14,15^, a wider intrinsic QRS duration^7,13,16^, LBBB^13^, a history of AF^6,17^, and a chronic higher RV pacing burden^8^ could be risk factors for PICM. However, these variables, but for a medical history of CAD, were not risk factors for PICM in our analysis.

Although it has been the general opinion that RV pacing burden has detrimental effects on left ventricular function, the present study revealed a higher proportion of patients who underwent PMI because of SSS rather than AVB in the PICM group (Table 1). Additionally, there was no difference in the percentage of RV pacing between patients with PICM and no PICM (Table 2). Recently, it was reported that no significant difference in the development of severe LV dysfunction is observed among patients with SSS and AVB in a large cohort of pacemaker recipients with normal LVEF^20^. Sanchez et al. showed that developing HF was not associated with pacing mode, %VP, or ventricular lead localization in patients with SSS^21^. These findings suggested that PICM could be developed in patients with SSS regardless of RV pacing burden.

The patients with PICM had a more prevalent medical history of CAD and a lower eGFR than those without PICM in this study. The multivariate Cox hazard model analysis also showed that a medical history of CAD or stages 4–5 CKD defined as an eGFR < 30 mL/min/1.73 m^2^ were significant risk factors associated with PICM. The result indicated that the medical history of CAD could be an independent risk factor for PICM as a past study reported. Tayal et al. reported that PMI patients with an antecedent history of MI and CKD had an increased risk of HF^22^. However, there is no previous study reported an association between renal dysfunction and PICM^13^. To the best of our knowledge, this study is the first report to show that an eGFR ≤ 30 mL/min/1.73 m^2^ is an independent risk factor for PICM.

The frequencies of PICM have been reported to be 5.1–26.8% at follow-up, with a mean period of 1–15 years^23^. The incidence rate of PICM in our study was 29.2% during a mean of 1.6 years of follow-up, which was slightly higher than that in many previous studies. However, a previous study reported a 39% prevalence of PICM according to the same definition as this study^2^. From the other point of view, this difference in the incidence in current study could be explained by the high prevalence of severe CKD (eGFR ≤ 30 mL/min/1.73 m^2^) in patients with PICM, in other words, a total of 11.5% of patients had a low GFR < 30 ml/min/1.73 m^2^.

Renal impairment confers a high risk for poor cardiovascular outcomes^24^, increasing mortality in patients with HF^25^. In particular, moderate/severe renal impairment has a high risk of developing HF^26^. Various mechanistic insights have been proposed for this finding. Renal impairment can upregulate the renin–angiotensin–aldosterone system and enhance basal sympathetic nerve discharge, increasing pro-inflammatory factors and oxidative stress. Additionally, endothelial dysfunction^27^, exacerbation of underlying anemia, and worsening of LV hypertrophy and myocyte contractility are related to the incidence of HF and impairment of ventricular systolic function.

The high phosphorus status associated with CKD also promotes the calcification of cardiac vessels and valves, further accelerating a reduced LVEF^28^. Atherosclerosis and CKD affect cardiac function through their interaction that worsens with each other.

Metabolic causality has also been reported as the cause of PICM. Several studies have proposed that RV pacing can induce abnormal myocardial metabolism and altered regional perfusion, increase fibrosis, and cause myofibrillar disarray^29–31^. However, these possibilities have still not been proven.

Recently, Lin et al. suggested a mechanism of PICM by which intracellular lipid accumulation induced by pacing increases fibrosis in the LV myocardium in some animal models, including pigs and rats^32,33^. In addition, they showed that the inhibition of the liver X receptor/retinoid X receptor pathway, which regulates lipid metabolism, inflammation, and cholesterol to bile acid catabolism, was associated with pacing^32^.

Moreover, the lipid-lowering and pleiotropic effects of treatment with statins are attributed to preventing PICM and reducing HF hospitalization, cardiovascular death, and all-cause mortality in patients with AVB^33^. These results suggest a causative role of lipid accumulation in PICM. Even in non-pacemaker-implanted patients, such as patients with diabetes mellitus and obesity, abnormal intramyocardial lipid accumulation has been frequently observed owing to metabolic changes in the heart. In addition, cardiac lipid accumulation is positively correlated with cardiac dysfunction, which is called lipotoxic cardiomyopathy^34^. Taking these findings into consideration, we believe that cardiac metabolic modulation due to RV pacing plays an important role in the impairment of cardiac function.

Our results could help to identify patients who develop PICM before PMI. Severe renal dysfunction and a history of CAD could be independent risk factors for PICM. Therefore, patients with these risk factors require closer follow-up by TTE to avoid missing the appropriate timing for upgrading to cardiac resynchronization therapy. Furthermore, conduction system pacing, such as His-bundle pacing and left-bundle pacing, are alternative options in patients with AVB and high-risk factors when they undergo PMI.

## Limitations

Our study has some limitations. First, this was a retrospective study from a single center with a comparatively small number of participants, and it did not reflect direct causation. Second, measurement with TTE was performed among not all patients with pacemaker, and the time window for performing follow-up TTE was unclear. Our observational periods for the occurrence of PICM were not long term but medium term instead. Third, PMI procedures in the current study likely included some older materials and methods, and our data lacked information on the lead tip position. Finally, further studies regarding the effects of CAD and CKD on the incidence of PICM after PMI are required to confirm our findings.

## Conclusions

A past medical history of CAD and severe stage CKD, stage 4 or 5, at PMI are newly discovered risk factors of PICM. Therefore, intensive follow-up is required to detect a deterioration in the LVEF at an early stage.

### Supplementary Information

Supplementary Table 1.

## Data Availability

The datasets generated and analyzed during the current study are available from the corresponding author on reasonable request.
