# Supplementary material for: The estimated glomerular filtration rate predicts pacemaker-induced cardiomyopathy
Source: Sci Rep. 2023 Oct 2;13:16514. doi: 10.1038/s41598-023-43953-7 (PMC10545821; doi:10.1038/s41598-023-43953-7)
Supplement: Supplementary file 1 — Supplementary Table 1. [file 41598_2023_43953_MOESM1_ESM.docx]

**Supplementary Table 1. Distribution of right ventricular lead tip positions**

| **Right ventricular lead**  **tip positions** | **Total (n = 113)** | **Non-PICM (n = 80)** | **PICM (n = 33)** | ***P*** |
| --- | --- | --- | --- | --- |
| Apex (n, %) | 32 (28.3) | 24 (30.0) | 8 (24.2) | 0.54 |
| Septum (n, %) | 70 (61.9) | 48 (60.0) | 22 (66.7) | 0.51 |
| Left bundle branch area (n, %) | 3 (2.7) | 2 (2.5) | 1 (3.0) | 1.00 |
| His bundle (n, %) | 8 (7.1) | 6 (7.5) | 2 (6.1) | 1.00 |

PICM, pacemaker-induced cardiomyopathy.
